# Effect of dipeptidyl peptidase-4 inhibitors on postprandial glucagon level in patients with type 2 diabetes mellitus: A systemic review and meta-analysis

**DOI:** 10.3389/fendo.2022.994944

**Published:** 2022-10-12

**Authors:** Shangyu Chai, Ruya Zhang, Ye Zhang, Richard David Carr, Yiman Zheng, Swapnil Rajpathak, Linong Ji

**Affiliations:** ^1^ Merck Research Laboratories (MRL) Global Medical Affairs, Merck Sharp & Dohme (MSD) China, Shanghai, China; ^2^ Hatter Cardiovascular Institute, University College London, UK and Ulster University, Coleraine, United Kingdom; ^3^ Merck Research Laboratories, Merck & Co., Inc., Rahway, NJ, United States; ^4^ Department of Endocrinology, People’s Hospital of Peking University, Beijing, China

**Keywords:** dipeptidyl peptidase-4 inhibitor, glucagon, hyperglucagonemia, randomized controlled trials, meta-analysis

## Abstract

**Aims:**

Hyperglucagonemia occurs in the pathogenesis of type 2 diabetes mellitus (T2DM). In this meta-analysis, we summarized the effects of DPP4 inhibitors on glucagon levels in patients with T2DM.

**Materials and methods:**

Randomized controlled trials (RCTs) comparing the influence of DPP4 inhibitors on circulating glucagon levels with placebo or other oral antidiabetic drugs (OADs) in patients with T2DM were identified by searches of Medline (PubMed), Embase (Ovid), and CENTER (Cochrane Library). Only studies reporting changes in glucagon level presented as total area under the curve (AUC_glucagon_) using a meal or oral glucose tolerance test were included. Results were combined using a random-effects model that incorporated potential heterogeneity among the included studies.

**Results:**

A total of 36 RCTs with moderate to high quality were included. Overall, the numbers of T2DM patients included for the meta-analyses comparing DPP4 inhibitors with placebo and other OADs were 4266 and 1652, respectively. Compared to placebo, DPP4 inhibitors significantly reduced circulating glucagon levels (standard mean difference [SMD]: -0.32, 95% CI: -0.40 to -0.24, *P*<0.001; I^2 =^ 28%). Analysis of subgroups revealed that study characteristics had no significant effect on results, such as study design (parallel group or crossover), number of patients, mean patient age, proportion of men, baseline HbA1c, duration of diabetes, background therapy, treatment duration, or methods for glucagon measurement (all *P* for subgroup differences >0.05). Moreover, DPP4 inhibitors significantly reduced glucagon levels compared to other OADs (SMD: -0.35, 95% CI: -0.53 to -0.16, *P*<0.001; I^2^ = 66%), and the reduction in glucagon was greater in comparison with insulin secretagogues than in comparison with non-insulin secretagogues (*P* for subgroup difference =0.03).

**Systematic review registration:**

https://inplasy.com/, identifier INPLASY202280104.

**Conclusions:**

DPP4 inhibitors are effective at reducing the circulating postprandial glucagon level in T2DM patients.

## Introduction

Currently, type 2 diabetes mellitus (T2DM) is one of the key factors contributing to morbidity and mortality worldwide (1, 1). It has conventionally been believed that insulin resistance and impaired insulin secretion are the key mechanisms underlying T2DM development (2, 3). Despite this common knowledge, abnormally elevated serum levels of glucagon (hyperglucagonemia) also contribute to diabetes pathogenesis (4–6), which may reflect α-cell dysfunction within the pancreatic islets. Clinical discussions often focus on insulin, but glucagon has an equally important role to play in understanding T2DM (7). Actually, all types of poorly controlled diabetes are associated with hyperglucagonemia (8). Therefore, treatment targeting hyperglucagonemia may also become an established antidiabetic strategy (9). There are several potential mechanisms for hyperglucagonemia in T2DM, including β-cell dysfunction, disturbances in α-cell/β-cell interplay, and dysfunctional incretin effect (10–13). Knowing the role of glucagon is crucial to appreciating differences in glucose-lowering therapies’ mechanisms of action. Amongst oral anti-diabetic drugs (OADs), dipeptidyl-peptidase 4 (DPP-4) inhibitors are a well-applied class of glucose-lowering medications that function by inhibiting the DPP-4–induced degradation of incretin hormone glucagon-like peptide-1 (GLP-1) and glucose-dependent insulinotropic polypeptide (GIP). The benefits of DPP-4 inhibitors in T2DM are not only limited to their regulation of insulin secretion in a glucose-dependent manner, but also include their efficacies in attenuating β-cell loss and improving glycemic durability (14, 15). Moreover, through increasing endogenous levels of the incretin hormones (10, 16), DPP-4 inhibitors have been suggested to suppress endogenous glucagon production in T2DM.

Compared with insulin, our understanding, research and detection methods for glucagon are not so well established. To the best of our knowledge, no consensus or gold standard has been reached regarding the optimal methods for measuring the serum glucagon concentration. In spite of the fact that plasma glucagon levels are not used in clinical stratification of diabetes treatment, health care providers may gain clinical insight by understanding how to control plasma glucagon levels pharmacologically in T2DM patients. In patients with T2DM, glucagon levels typically rise during fasting and then fail to decrease appropriately or even rise during oral glucose tolerance testing (OGTT) or after ingestion of a carbohydrate-rich meal, leading to undesirably high plasma glucagon with hyperglycemia. Typically, the effects of antidiabetic treatment on the postprandial glucagon level are measured by the changes in glucagon total area under the curve (AUC_glucagon_) using a meal tolerance test (MTT) or a standard OGTT (17–19). Although there have been few small-scale randomized controlled trials (RCTs) evaluating the effect of DPP-4 inhibitors on plasma glucagon levels in patients with type 2 diabetes (20–55), little is known about the summarized efficacy of DPP4 inhibitors on AUC compared to placebo. There are also other oral glucose-lowering drug classes that affect glucagon secretion (positively or negatively), including sulfonylureas and sodium-glucose cotransporter 2 inhibitors (SGLT2is). Therefore, the summarized efficacy of DPP4 inhibitors on AUC_glucagon_ compared to other OADs also seems interesting. To the best of our knowledge, no systematic review and meta-analysis has been published to date regarding the influence of DPP4 inhibitors on AUC_glucagon_ in patients with T2DM. Accordingly, the aim of this study was to examine systematically the influence of DPP4 inhibitors on AUC_glucagon_ in T2DM patients by performing a meta-analysis of RCTs.

## Methods

This study adhered to PRISMA (Preferred Reporting Items for Systematic Reviews and Meta-Analyses) (56) and Cochrane Handbook (57) guidelines during its design and implementation. The protocol of the meta-analysis was registered at the International Platform of Registered Systematic Review and Meta-analysis Protocols (INPLASY, https://inplasy.com/) with the registration number of INPLASY202280104. The PRISMA 2020 Checklist has been provided in the **Supplementary Material**.

### Search strategy

In order to search Medline (PubMed), Embase (Ovid), and CENTER (Cochrane Library), the following strategies were used: (1) “DPP4” OR “DPP-4” OR “dipeptidyl peptidase-4 inhibitors” OR “sitagliptin” OR “vildagliptin” OR “linagliptin” OR “saxagliptin” OR “alogliptin” OR “dutogliptin” OR “aemigliptin” OR “anagliptin” OR “teneligliptin” OR “trelagliptin” OR “omarigliptin” OR “gemigliptin” OR “evogliptin”; (2) “α cell” OR “α-cell” OR “glucagon” OR “alpha cell” OR “islet” OR “hormone” OR “hormonal” OR “meal” OR “prandial” OR “postprandial” OR “Oral Glucose Tolerance Test” OR “OGTT”; and (3) “random” OR “randomized” OR “randomised” OR “randomly”. Only studies including human subjects were considered. As part of the final database search, references to related reviews and original articles were also searched. The final database search was carried out on August 25, 2022.

### Study selection

We included studies that met the following criteria: (1) English-language articles with full-length content; (2) RCTs with parallel groups or crossovers; (3) Adults with T2DM were randomly assigned to DPP4 inhibitors or placebo groups, or other OADS, for treatment; and (4) reported the changes of AUC_glucagon_ from baseline after treatment utilizing MTT or OGTT in participants in the interventional and control arms. In this review, we included studies with patients who are drug-nave or with T2DM patients who are receiving background OAD therapy. However, studies including T2DM patients on concurrent antidiabetic injection treatment, such as insulin and GLP-1 receptor agonists (GLP-1RAs) were excluded from the current meta-analysis. Our meta-analysis did not include studies that included patients treated with single-dose or single-day DPP4 inhibitors, since we weren’t planning to evaluate the acute effects of DPP4 inhibitors on circulating glucagon. In addition, non-randomized studies, studies including non-T2DM patients, or those without a measurement of AUC_glucagon_ during MTT or OGTT setting were also excluded.

### Data collection and quality evaluation

Database searches, data collection, and quality assessment were carried out by two authors independently. Discussions with the corresponding author were conducted if disagreements occurred. We collected data of study information (first author, publication year, and study country), study design (blind or open-label, crossover or parallel group), patient information (number of patients, mean age, sex, baseline hemoglobin A1c [HbA1c], and T2DM duration), details of background antidiabetic treatments, drugs and doses of DPP4 inhibitors used, regimens of controls, treatment durations, and methods for circulating glucagon measurement. The Cochrane Risk of Bias Tool was used to determine the quality of the included RCTs (57) according to the following aspects: assigning random sequences; concealing allocations; blinding participants and personnel; blinding outcomes assessors; incomplete outcomes data; and selective outcome reporting.

### Statistical analysis

The effects of DPP4 inhibitors on circulating glucagon levels compared to controls in T2DM patients were presented as a standard mean difference (SMD) with 95% confidence interval (CI) because the methods and durations for measuring AUC_glucagon_ varied among the included RCTs. Heterogeneity was assessed using Cochrane’s Q test (58). It was also calculated the I^2^ statistic, and an I^2^ >50% indicates significant heterogeneity. A random-effects model was used when calculating pooled analyses, since it incorporates potential heterogeneity and provides more generalized results (57). Analyses of sensitivity were conducted by excluding one study at a time from the meta-analysis to evaluate the effect of each study on the pooled results (57). Additionally, sensitivity analyses limited to studies with FDA-approved DPP4 inhibitors and dosages were also performed, including sitagliptin 100 mg once daily, saxagliptin 5 mg once daily, linagliptin 5 mg once daily, and alogliptin 25 mg once daily. Analysis of predefined subgroups was conducted to determine whether study characteristics could influence the results, including study characteristics such as study design (parallel group or crossover), number of patients, mean patient age, proportion of men, baseline HbA1c, duration of diabetes, background therapy, treatment duration, and methods for glucagon measurement. For continuous variables, medians were selected as cutoffs for defining of subgroups. For a meta-analysis comparing DPP4 inhibitors and other OADs, subgroup analyses were performed according to whether the OADs taken by controls were insulin secretagogues or non-insulin secretagogues. An evaluation of publication bias was conducted *via* visual inspection of funnel plots and Egger’s regression asymmetry test (59). For studies including multiple dose groups of DPP4 inhibitors, the shared control groups were equally split and included as independent comparisons to overcome a unit-of-analysis error, according to the instruction of Cochrane’s Handbook (57). Differences for which *P*<0.05 were considered statistically significant. Statistical analyses were conducted using the RevMan (Version 5.1; Cochrane, Oxford, UK) software.

## Results

### Literature search

**Figure 1:**
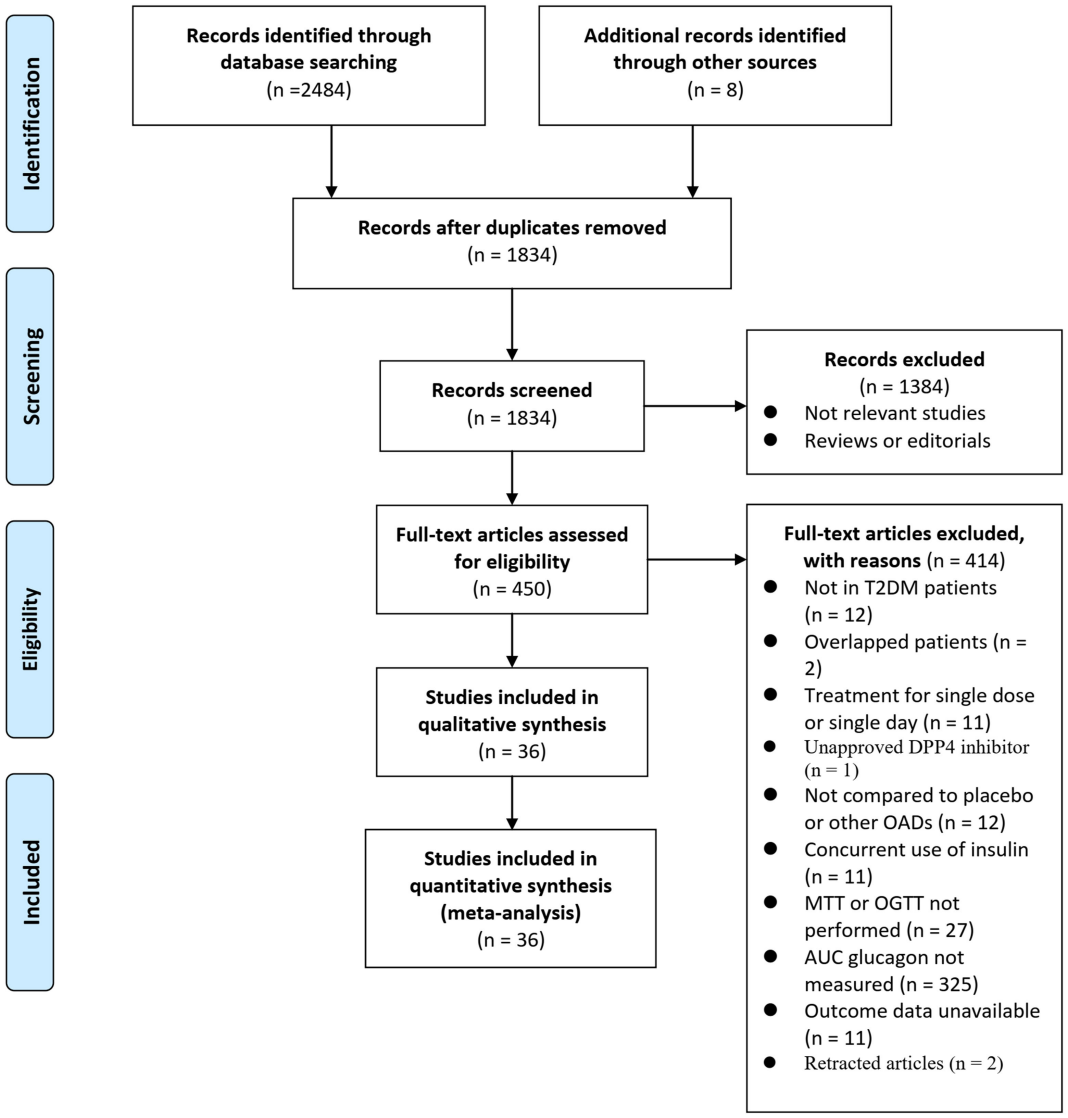
Flowchart of literature search.

**Figure 1** illustrates the process of searching databases and identifying studies. Briefly, database searches yielded 2492 articles, and 1834 were retrieved after the duplicate records were excluded. Thirteen hundred eighty-four articles were subsequently excluded based on titles and abstracts, primarily because they were unrelated to the goal of the meta-analysis. Then, 414 articles out of the 450 that received full-text reviews were further excluded for the reasons illustrated in **Figure 1**. Finally, 36 RCTs (20–55) were deemed to be eligible for the meta-analysis.

### Study characteristics and data quality

An overview of the included studies can be found in **Table 1**. Overall, 23 studies compared DPP4 inhibitors with placebo (20–22, 25–27, 30–42, 44–47). Eight studies compared DPP4 inhibitors with other OADs (28, 48–55), and the other five studies included both comparisons with placebo and other OADs (23, 24, 28, 29, 43).

**Table 1:** Characteristics of the included studies.

Comparisons between DPP4 inhibitors and placebo treatment												
Study	Country	Design	Patient number	Mean age	Male	Baseline HbA1c	T2DM duration	Background treatment	Intervention	Control	Treatment duration	Postprandial glucagon measuring
				year	%	%	years				days	
He 2007	USA	R, DB, PC, CO	12	53.5	46.2	NR	7.2	Treatment naïve or with OAD	Vildagliptin 10mg, 25mg Bid	Placebo	28	MTT-4h, RIA
Vella 2007	USA	R, DB, PC, CO	14	53.1	35.7	6.1	NR	Treatment naïve or with OAD (metformin etc.)	Vildagliptin 50mg Bid	Placebo	10	MTT-5h, RIA
Defronzo 2009	USA	R, DB, PC	401	54.6	50.1	8	6.5	Metformin	Saxagliptin 2.5mg, 5mg Qd	Placebo	147	OGTT-3h, RIA
Ahren 2009	Sweden	R, DB, PC, CO	25	65.5	88	6.3	5.6	Treatment naïve	Vildagliptin 50mg Bid	Placebo	28	MTT-2h, RIA
Rosenstock 2009	USA	R, DB, PC	201	53.6	52.5	7.9	2.6	Treatment naïve	Saxagliptin 2.5mg, 5mg Qd	Placebo	168	OGTT-2h, RIA
Jadzinsky 2009	Argentina	R, DB, PC	269	51.9	50.6	9.4	1.9	Metformin	Saxagliptin 5mg Qd	Placebo	168	OGTT-3h, RIA
Kikuchi 2009	Japan	R, DB, PC	78	59	67	7.4	5.3	Treatment naïve	Vildagliptin 10mg, 25mg, 50mg Bid	Placebo	84	MTT-2h, RIA
Hollander 2009	USA	R, DB, PC	384	54	49.5	8.3	5.2	Thiazolidinedione	Saxagliptin 2.5mg, 5mg Qd	Placebo	168	OGTT-3h, RIA
Iwamoto 2010	Japan	R, DB, PC	129	59.7	62.3	7.6	5.5	Treatment naïve	Sitagliptin 25mg, 50mg, 100mg Qd	Placebo	84	MTT-2h, RIA
Seino 2011a	Japan	R, DB, PC	230	62.2	61.7	8	7.7	Voglibose	Alogliptin 12.5mg, 25mg Qd	Placebo	84	MTT-2h, RIA
Seino 2011b	Japan	R, DB, PC	318	58.9	73.1	7.9	6.6	Treatment naïve	Alogliptin 6.25mg, 12.5mg, 25mg Qd	Placebo	84	MTT-2h, RIA
Henry 2011	USA	R, DB, PC	36	55.6	38.9	6.8	3.2	Treatment naïve	Saxagliptin 5mg Qd	Placebo	84	OGTT-5h, RIA
Tremblay 2011	Canada	R, DB, PC, CO	36	58.1	83.3	6.8	NR	Metformin	Sitagliptin 100mg Qd	Placebo	42	MTT-8h, RIA
Rauch 2012	Germany	R, DB, PC	80	NR	NR	7.3	NR	Treatment naïve or with OAD	Linagliptin 5mg Qd	Placebo	28	MTT-2h, RIA
Eto 2012	Japan	R, DB, PC	99	57.2	84.8	8.3	6.8	Treatment naïve	Teneligliptin 10mg, 20mg Qd	Placebo	28	MTT-2h, RIA
Bunck 2012	The Netherlands	R, DB, PC	59	57.2	59.3	6	1	Treatment naïve	Vildagliptin 100mg Qd	Placebo	350	MTT-2h, RIA
Alba 2013	USA	R, DB, PC	94	54.9	57.1	7.9	2.4	Treatment naïve or with OAD	Sitagliptin 100mg Qd	Placebo	147	MTT-3h, RIA
Kadowaki 2013	Japan	R, DB, PC	324	58.2	65.7	7.8	6.2	Treatment naïve or with OAD	Teneligliptin 10mg, 20mg, 40 mg Qd	Placebo	84	MTT-2h, RIA
Sjostrand 2014	China	R, DB, PC	431	52.9	50.2	7.9	3.7	Metformin	Saxagliptin 5mg Qd	Placebo	168	MTT-3h, RIA
Van Raalte 2014	The Netherlands	R, DB, PC	49	58.9	71.4	6.7	NR	Metformin and other OAD	Alogliptin 25mg Qd	Placebo	112	MTT-8h, RIA
Vardarli 2014	Germany	R, DB, PC, CO	20	59	80	7	5	Treatment naïve or with OAD	Sitagliptin 100mg Qd	Placebo	5	OGTT-4h, RIA
Kadowaki 2014	Japan	R, DB, PC	194	59.4	66	8.4	8.8	Glimepiride	Teneligliptin 20mg Qd	Placebo	84	MTT-2h, RIA
Hansen 2014	Sweden	R, DB, PC	312	54	47	8.9	7.3	Dapagliflozin and metformin	Saxagliptin 5mg Qd	Placebo	168	MTT-3h, RIA
Nishimura 2016	Japan	R, OL	38	63.4	72.5	7.7	7.6	Repaglinide	Sitagliptin 100mg Qd	No treatment	168	MTT-3h, RIA
Forst 2017	Germany	R, DB, PC	44	63.8	90.9	8.1	9.7	Empagliflozin	Linagliptin 5mg Qd	Placebo	28	MTT-3h, RIA
Ahn 2017	Korea	R, DB, PC, CO	10	56.5	30	7.2	11.8	Treatment naïve or with OAD	Gemigliptin	Placebo	28	MTT-4h, RIA
Farngren 2018	Sweden	R, DB, PC, CO	28	73.6	61	6.9	9.2	Metformin	Sitagliptin 100mg Qd	Placebo	28	MTT-2h, RIA
Dou 2018	China	R, DB, PC	231	50.1	66.5	9.4	0.8	Metformin	Saxagliptin 5mg Qd	Placebo	168	MTT-3h, RIA
Comparisons between DPP4 inhibitors and other OADs												
Ahren 2010	Sweden	R, DB	259	57.5	53.4	7.3	5.7	Metformin	Vildagliptin 50mg Bid	Glimepiride 6mg Qd (max)	657	MTT-2h, RIA
Seino 2011b	Japan	R, DB	326	58.9	71.9	7.9	6.5	Treatment naïve	Alogliptin 6.25mg, 12.5mg, 25mg Qd	Voglibose 0.2mg Tid	84	MTT-2h, RIA
Alba 2013	USA	R, DB	95	53.4	51.4	8	2.4	Treatment naïve or with OAD	Sitagliptin 100mg Qd	Pioglitazone 30mg Qd	147	MTT-3h, RIA
Okada 2013	Japan	R, OL	34	65.5	38.2	7.8	8.2	Treatment naïve or with OAD	Sitagliptin 50mg Qd	Miglitol 50mg Tid	70	MTT-2h, RIA
Forst 2014	Germany	R, OL	39	64	69.2	7.4	7.8	Metformin	Linagliptin 5mg Qd	Glimepiride 4mg Qd (max)	84	MTT-5h, RIA
Vardarli 2014	Germany	R, DB, CO	20	59	80	7	5	Treatment naïve or with OAD	Sitagliptin 100mg Qd	Metformin 500mg Bid~Qid	5	OGTT-4h, RIA
Hansen 2014	Sweden	R, DB	306	55	49	8.9	7.5	Metformin	Saxagliptin 5mg Qd	Dapagliflozin 10mg Qd	168	MTT-3h, RIA
Akiyama 2016	Japan	R, OL, CO	16	66	62.5	6.6	11.5	Pioglitazone or metformin	Sitagliptin 50 and 100mg Qd each for a week	Mitiglinide 10mg Tid	14	OGTT-3h, RIA
Xiao 2016	China	R, OL	41	68.9	56.1	7.2	Newly diagnosed	Metformin	Sitagliptin 100mg Qd	Glimepiride 1-4mg Qd	168	OGTT-2h, ELISA
Alsalim 2018	Sweden	R, DB, CO	28	63	71.4	6.8	5.8	Metformin	Vildagliptin 50mg Bid	Dapagliflozin 10mg Qd	14	MTT-4h, RIA
Dou 2018	China	R, DB	229	49.8	66.1	9.5	0.7	Treatment naïve	Saxagliptin 5mg Qd	Metformin 500-2000 mg per day	168	MTT-3h, RIA
Scott 2018	New Zealand	R, DB	173	67.1	57.9	7.8	10.6	Metformin	Sitagliptin 100mg Qd	Dapagliflozin 5-10mg Qd	168	MTT-2h, RIA
Nakagawa 2019	Japan	R, OL	22	61.2	40.9	7	5.3	Treatment naïve or with OAD	Anagliptin 100mg Bid	Metformin 500mg Bid	28	MTT-3h, LC-HRMS

DPP4, dipeptidyl-peptidase 4; HbA1c, glycosylated hemoglobin; T2DM, type 2 diabetes mellitus; R, randomized; DB, double blind; OL, open label; PC, placebo-controlled; CO, crossover; NR, not reported; OAD, oral antidiabetic drug; Bid, twice daily; Qd, once daily; Tid, three times daily; Qid, four times daily; MTT, meal tolerance test; OGTT, oral glucose tolerance test; RIA, radioimmunoassay; LC-HRMS, liquid chromatography-high resolution mass spectrometry.

Accordingly, a total of 28 RCTs were available for the meta-analysis comparing the influence of DPP4 inhibitors with placebo on postprandial glucagon (20–47). The characteristics of these studies are presented in the upper panel of **Table 1**. Briefly, these studies were all RCTs including T2DM patients which were published between 2007 and 2018. Seven of them were crossover studies (20, 24, 25, 27, 31, 37, 45), whilst the remaining studies were parallel-group RCTs. The mean ages of the patients varied between 51 and 74 years. Various DPP4 inhibitors were used among these studies, such as vildagliptin, saxagliptin, linagliptin, sitagliptin, alogliptin, teneligliptin, and gemigliptin, whilst placebo was used as the control in all of the included RCTs except one study which received no treatment (42). The treatment durations varied between 5 and 350 days, and circulating postprandial glucagon was measured with radioimmunoassay in MTT/OGTT settings. Using Cochrane’s Risk of Bias Tool, **Table 2** provides a detailed analysis of the included RCTs.

**Table 2:** Details of study quality evaluation *via* the Cochrane’s Risk of Bias Tool.

Comparisons between DPP4 inhibitors and other OADs							
Study	Random sequence generation	Allocation concealment	Blinding of participants	Blinding of outcome assessment	Incomplete outcome data addressed	Selective reporting	Other sources of bias
He 2007	Unclear	Unclear	Low	Low	Low	Low	Low
Vella 2007	Unclear	Low	Low	Low	Low	Low	Low
Defronzo 2009	Unclear	Unclear	Low	Low	Low	Low	Low
Ahren 2009	Unclear	Unclear	Low	Low	Low	Low	Low
Rosenstock 2009	Unclear	Unclear	Low	Low	Low	Low	Low
Jadzinsky 2009	Unclear	Unclear	Low	Low	Low	Low	Low
Kikuchi 2009	Unclear	Unclear	Low	Low	Low	Low	Low
Hollander 2009	Unclear	Unclear	Low	Low	Low	Low	Low
Iwamoto 2010	Unclear	Unclear	Low	Low	Low	Low	Low
Seino 2011a	Low	Unclear	Low	Low	Low	Low	Low
Seino 2011b	Unclear	Unclear	Low	Low	Low	Low	Low
Henry 2011	Unclear	Unclear	Low	Low	Low	Low	Low
Tremblay 2011	Unclear	Unclear	Low	Low	Low	Low	Low
Rauch 2012	Unclear	Unclear	Low	Low	Low	Low	Low
Eto 2012	Unclear	Unclear	Low	Low	Low	Low	Low
Bunck 2012	Unclear	Unclear	Low	Low	Low	Low	Low
Alba 2013	Unclear	Unclear	Low	Low	Low	Low	Low
Kadowaki 2013	Unclear	Unclear	Low	Low	Low	Low	Low
Sjostrand 2014	Unclear	Unclear	Low	Low	Low	Low	Low
Van Raalte 2014	Unclear	Unclear	Low	Low	Low	Low	Low
Vardarli 2014	Unclear	Unclear	Low	Low	Low	Low	Low
Kadowaki 2014	Unclear	Unclear	Low	Low	Low	Low	Low
Hansen 2014	Unclear	Unclear	Low	Low	Low	Low	Low
Nishimura 2016	Low	Unclear	High	High	Low	Low	Low
Forst 2017	Unclear	Unclear	Low	Low	Low	Low	Low
Ahn 2017	Unclear	Unclear	Low	Low	Low	Low	Low
Farngren 2018	Unclear	Unclear	Low	Low	Low	Low	Low
Dou 2018	Unclear	Unclear	Low	Low	Low	Low	Low

Comparisons between DPP4 inhibitors and other OADs							
Study	Random sequence generation	Allocation concealment	Blinding of participants	Blinding of outcome assessment	Incomplete outcome data addressed	Selective reporting	Other sources of bias
Ahren 2010	Low	Low	Low	Low	Low	Low	Low
Seino 2011b	Unclear	Unclear	Low	Low	Low	Low	Low
Alba 2013	Unclear	Unclear	Low	Low	Low	Low	Low
Okada 2013	Unclear	Unclear	High	High	Low	Low	Low
Forst 2014	Unclear	Unclear	High	High	Low	Low	Low
Vardarli 2014	Unclear	Unclear	Low	Low	Low	Low	Low
Hansen 2014	Unclear	Unclear	Low	Low	Low	Low	Low
Akiyama 2016	Unclear	Unclear	High	High	Low	Low	Low
Xiao 2016	Unclear	Unclear	High	High	Low	Low	Low
Alsalim 2018	Unclear	Unclear	Low	Low	Low	Low	Low
Dou 2018	Unclear	Unclear	Low	Low	Low	Low	Low
Scott 2018	Unclear	Unclear	Low	Low	Low	Low	Low
Nakagawa 2019	Unclear	Unclear	High	High	Low	Low	Low

A total of 13 studies compared circulating glucagon levels in T2DM patients treated with a DPP4 inhibitor or other OAD (23, 24, 28, 29, 43, 48–55). Vildagliptin, sitagliptin, linagliptin, saxagliptin, alogliptin, and anagliptin were used as treatments, while glimepiride, voglibose, pioglitazone, dapagliflozin, miglitol and metformin were used as controls. The mean ages of the patients varied between 51 and 69 years. The follow-up durations varied from 5–657 days. Circulating glucagon levels were measured by radioimmunoassay (23, 24, 28, 29, 43, 48–51, 53, 54), enzyme-linked immunosorbent assay (ELISA) (55), or liquid chromatography-high resolution mass spectrometry (52) in a MTT/OGTT setting. A detailed risk of bias assessment of the included RCTs can also be found in **Table 2**.

### Comparisons between DPP4 inhibitors and placebo on circulating postprandial glucagon

**Figure 2:**
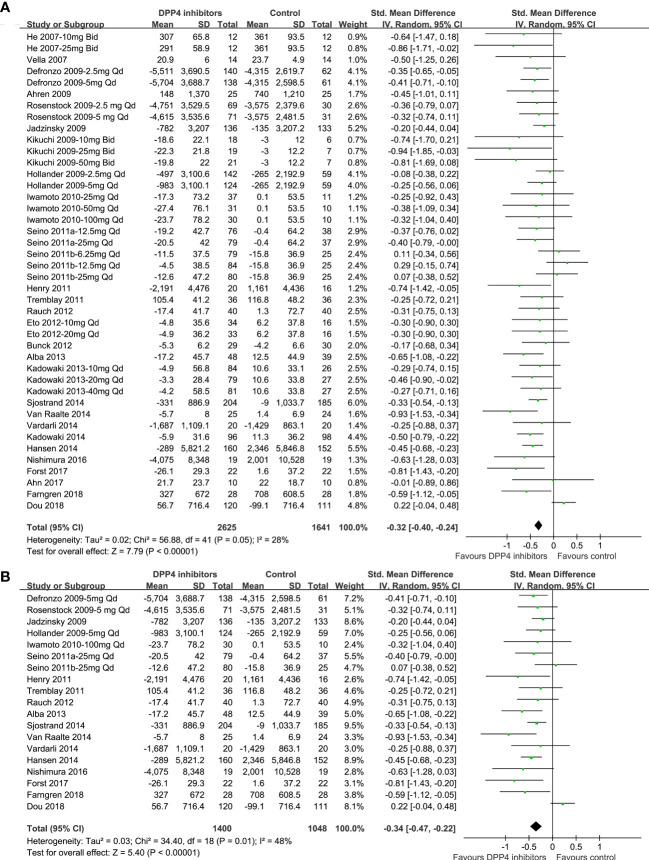
Forest plots for the meta-analysis comparing the effects of DPP4 inhibitors and placebo on circulating glucagon levels in T2DM patients. **(A)** Forest plots for the overall meta-analysis; and **(B)** forest plots for the sensitivity analyses limited to studies with FDA-approved DPP4 inhibitors and dosages.

Because 10 studies reported data according to multiple dosages of DPP4 inhibitors separately (20–23, 26, 30, 35, 38, 41, 46), these datasets were included into the meta-analysis independently. Accordingly, 42 datasets from 28 RCTs were available comparing the effects of DPP4 inhibitors and placebo on circulating glucagon levels in T2DM patients (20–47). Studies included in this review showed mild heterogeneity (P for Cochrane’s Q test =0.05, I^2 =^ 28%). Pooled results showed that compared to placebo treatment, DPP4 inhibitors significantly reduced the circulating postprandial glucagon level in patients with T2DM (AUC_glucagon_: SMD=-0.32, 95% CI: -0.40 to -0.24, *P*<0.001; **Figure 2A**). The results were not significantly affected by excluding one dataset at a time during the sensitivity analysis (SMD: -0.31 ~ -0.34, all *P*<0.05). Sensitivity analyses limited to studies with FDA-approved DPP4 inhibitors and dosages showed consistent results (SMD = -0.34, 95% CI: -0.47 to -0.22, *P*<0.001; **Figure 2B**). Subgroup analyses showed that the results were not significantly affected by study characteristics such as study design (parallel group or crossover), number of patients, mean patient age, proportion of men, baseline HbA1c, duration of diabetes, background therapy, treatment duration, or methods for glucagon measurement (all *P* for subgroup difference >0.05; **Table 3**).

**Table 3:** Subgroup analysis for comparing DPP4 inhibitors with placebo treatment on circulating glucagon.

	Datasets	SMD (95% CI)	P for subgroup effect	I^2^	P for subgroup difference
Design					
Crossover	8	-0.42 [-0.65, -0.20]	< 0.001	0%	
Parallel group	34	-0.31 [-0.40, -0.22]	< 0.001	37%	0.37
Mean age (years)					
≤ 55	19	-0.29 [-0.40, -0.19]	< 0.001	35%	
> 55	22	-0.36 [-0.49, -0.23]	< 0.001	25%	0.47
Male (%)					
≤ 65	21	-0.33 [-0.42, -0.24]	< 0.001	0%	
> 65	20	-0.32 [-0.48, -0.16]	< 0.001	57%	0.89
Baseline HbA1c (%)					
≤ 7.0	8	-0.45 [-0.65, -0.25]	< 0.001	0%	
7.0~8.0	23	-0.32 [-0.41, -0.23]	< 0.001	0%	
> 8.0	9	-0.26 [-0.45, -0.07]	0.007	65%	0.37
T2DM duration (years)					
≤ 6	17	-0.29 [-0.41, -0.17]	< 0.001	32%	
> 6	21	-0.35 [-0.46, -0.24]	< 0.001	21%	0.46
Background treatment					
Drug naïve	16	-0.24 [-0.40, -0.09]	0.002	16%	
With OAD	16	-0.34 [-0.46, -0.21]	< 0.001	53%	
Drug naïve or with OAD	10	-0.41 [-0.58, -0.24]	< 0.001	0%	0.38
Treatment duration (days)					
≤ 84	28	-0.34 [-0.44, -0.24]	< 0.001	2%	
> 84	14	-0.30 [-0.43, -0.17]	< 0.001	54%	0.66
Glucagon measuring					
MTT	33	-0.34 [-0.45, -0.23]	< 0.001	38%	
OGTT	9	-0.28 [-0.39, -0.16]	< 0.001	0%	0.44

DPP4, dipeptidyl-peptidase 4; HbA1c, glycosylated hemoglobin; T2DM, type 2 diabetes mellitus; OAD, oral antidiabetic drug; MTT, meal tolerance test; OGTT, oral glucose tolerance test; SMD, standard mean difference; CI, confidence interval.

### Comparisons between DPP4 inhibitors and other OADs on circulating postprandial glucagon

**Figure 3:**
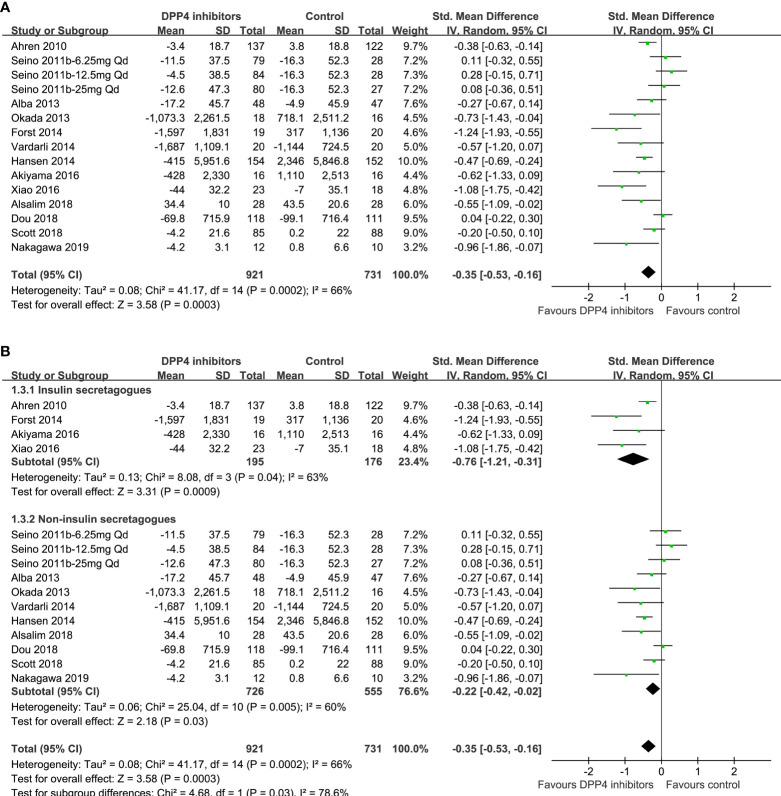
Forest plots for the meta-analysis comparing the effects of DPP4 inhibitors and other OADs on circulating glucagon levels in T2DM patients. **(A)** Forest plots for the overall meta-analysis; and **(B)** forest plots for the subgroup analyses according to the OADs given to controls.

One study reported data according to multiple dosages of DPP4 inhibitors separately (23), and these three datasets were included into the meta-analysis independently. Overall, the pooled results of 15 datasets from 13 RCTs (23, 24, 28, 29, 43, 48–55) showed that compared with other OADs, DPP4 inhibitors significantly reduced the circulating postprandial glucagon level in patients with T2DM (AUC_glucagon_: SMD=-0.35, 95% CI: -0.53 to -0.16, *P*<0.001; I^2 =^ 66%; **Figure 3A**). Sensitivity analysis by excluding one dataset at a time did not significantly change the results (SMD: -0.30 ~ -0.39, all *P*<0.05). Subgroup analyses showed that compared with either insulin secretagogues or non-insulin secretagogues, DPP4 inhibitors still significantly reduce glucagon levels in T2DM patients, and the reduction of glucagon was more remarkable compared with insulin secretagogues (SMD: -0.76, 95% CI: -1.21 to -0.31, *P*<0.001) than with non-insulin secretagogues (SMD: -0.22, 95% CI: -0.42 to -0.02, *P*=0.03; *P* for subgroup difference =0.03; **Figure 3B**).

### Publication bias

**Figure 4:**
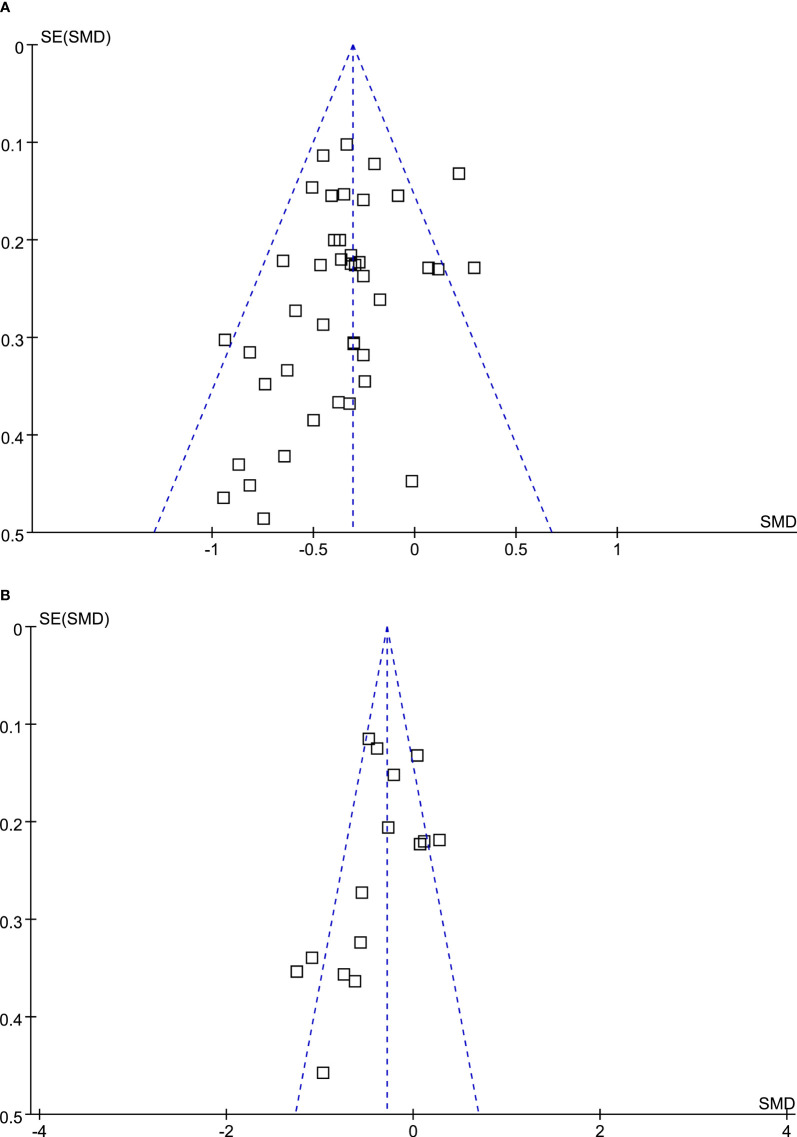
Funnel plots for the meta-analysis evaluating the influences of DPP4 inhibitors on circulating glucagon in T2DM patients. **(A)** Funnel plots for the meta-analysis comparing the effects of DPP4 inhibitors and placebo; and **(B)** funnel plots for the meta-analysis comparing the effects of DPP4 inhibitors and other OADs.

The funnel plots for the meta-analyses comparing DPP4 inhibitors with placebo and other OADs were symmetrical, suggesting low-risk of publication biases (**Figures 4A, B** **)**. Egger’s regression tests also suggested low risk of publication biases (*P*=0.167 and 0.156, respectively).

## Discussion

Based on a pooled analysis of RCT results, this meta-analysis concluded that DPP4 inhibitors are effective at reducing postprandial glucagon levels in T2DM patients compared to placebo, as evidenced by a significantly reduced AUC_glucagon_ in MTT/OGTT settings in patients allocated to the DPP4 inhibitor arm. Moreover, multiple sensitivity and subgroup analyses confirmed the stability of the findings, which were not significantly driven by any one of the included studies or significantly affected by predefined study characteristics, such as study design (parallel group or crossover), number of patients, mean patient age, proportion of men, baseline HbA1c, duration of diabetes, background therapy, treatment duration, or methods for glucagon measurement. In addition, the pooled results of 13 eligible RCTs showed that DPP4 inhibitors may also significantly reduce the circulating postprandial glucagon level in T2DM patients as compared to other OADs. Researchers have suggested that factors originating from nutrient stimulation of the digestive tract may play a key role in this process, namely, that postprandial glucagon production may be gut-derived, not originating from the pancreas (60, 61). Therefore, we divided other OADs into two groups for subgroup analysis. The results of this subgroup analysis showed that DPP4 inhibitors significantly reduced glucagon levels in T2DM patients compared to both the insulin and non-insulin secretagogues, separately, and the reduction in glucagon level was more noteworthy compared to that achieved with insulin secretagogues than with non-insulin secretagogues. Taken together, these results indicate that DPP4 inhibitors are effective at reducing postprandial glucagon levels in T2DM patients, which may be an additional mechanism underlying their benefits in patients with T2DM.

As far as we know, this is the first meta-analysis examining how DPP4 inhibitors affect circulating postprandial glucagon levels in people with T2DM. Unlike pharmacological studies in healthy volunteers with a single dose of the medication, we only included studies with T2DM patients who were treated for a stable period with DPP4 inhibitors, aiming to show that the possible glucagon-lowering efficacy of DPP4 inhibitors is clinically relevant. Because the relevant data on glucagon levels after fasting is limited and the circulating level of glucagon is variable according to feeding status, we chose AUC_glucagon_ using standard MTT/OGTT as the measurement rather than plasma glucagon level at a certain time to comprehensively reflect the dynamic status of postprandial glucagon (62). Studies including patients with concurrent insulin, GLP-1RAs or pramlintide injections were also excluded, because the imbalance of these treatments between groups may significantly affect the circulating glucagon levels (63, 64). These methodological considerations minimized the possible confounding effects of other clinical factors on the level of glucagon. The glucagon-suppressing effect of DPP4 inhibitors in T2DM patients observed in this meta-analysis could be explained by the pharmacological mechanisms of the drugs and confirmed that DPP4 inhibitors improve glycemic control at least partially *via* influencing glucagon levels. As part of its biological function, GLP-1 stimulates insulin secretion and inhibits glucagon action when secreted by the small intestine’s L-cells. By inhibiting the DPP-4–induced degradation of GLP-1, DPP4 inhibitors enhance the inhibitory effect of GLP-1 on glucagon, which may be more remarkable in a hyperglucagonaemic state in T2DM patients (64). Interestingly, glucose-dependent insulinotropic polypeptide also increases glucagon secretion under hypoglycemic and euglycemic conditions, but not under hyperglycemic conditions (65–67). This specific mechanism is rarely reported for other OADs, which is also consistent with our findings that DPP4 inhibitors significantly reduced circulating postprandial glucagon levels in T2DM patients as compared to other OADs. In view of the importance of hyperglucagonemia in the pathogenesis of T2DM, the results of this study highlight the additional benefits of glucagon suppression by DPP4 inhibitors compared to other commonly used OADs. Interestingly, accumulating evidence from clinical studies has suggested that similar to DPP-4 inhibitors, GLP-1RAs substantially lower glucagon concentrations in both the fasting state and after a meal, thus reducing the hyperglucagonemia in patients with T2DM (68, 69). However, nearly all cardiovascular outcomes trials conducted with DPP4 inhibitors in T2DM patients so far have demonstrated a neutral effect on major adverse cardiovascular events (70, 71).

This meta-analysis has several strengths, including a rigorous literature review, strict inclusion and exclusion criteria, and robust results, comprehensive full-text review to include available related RCTs, and performance of multiple sensitivity analyses to confirm the stability and robustness of the findings. However, this study also has limitations. First, the optimal assessment method for circulating glucagon remains to be developed (72). Therefore, differences in the measurement methods for glucagon among the included studies may have contributed to the clinical heterogeneity of the meta-analysis, such as the different durations for MTT/OGTT and assays for plasma glucagon. In addition, the data used for this meta-analysis were study-level rather than individual patient-level. Subgroup analyses, therefore, should be interpreted cautiously. Large-scale RCTs or meta-analyses based on individual patient data may be considered to validate whether patient characteristics or concurrent medications may influence the potential glucagon-suppressing effect of DPP4 inhibitors. Besides, although hyperglucagonemia has been known to be involved in the pathogenesis and progression of T2DM, studies evaluating the significance of hyperglucagonemia in determination of glycemic control and prognosis in patients with T2DM are rare, at least partly because of the lack of standard methods for glucagon measuring in clinical practice. Future studies are also needed in this regard.

Overall, the results of this meta-analysis showed that DPP4 inhibitors are effective at reducing circulating postprandial glucagon levels in T2DM patients. In view of the importance of hyperglucagonemia in the pathogenesis of T2DM, these results highlight the additional effect of DPP4 inhibitors in patients with T2DM.

## Conclusions

In conclusion, this meta-analysis revealed the effectiveness of DPP4 inhibitors for reducing circulating postprandial glucagon levels in T2DM patients in comparison with placebo or other OADs. The results confirmed that DPP4 inhibitors improve glycemic control, in part, by affecting glucagon levels.

## Data availability statement

The original contributions presented in the study are included in the article/**Supplementary Material**. Further inquiries can be directed to the corresponding author.

## Author contributions

SC, YZ, and RC conceived, designed, or planned the study; SC collected data and conducted research; RZ and LJ performed or supervised analyses; RZ, SR and YMZ interpreted the results; SC wrote the initial paper; All authors provided substantive suggestions for revision or critically reviewed subsequent iterations of the manuscript, reviewed and approved final version of the paper, and for all aspects of the work in ensuring that questions related to the accuracy.

## Funding

This study received funding from MSD China. The funder had the following involvement in the study: study design, data collection and analysis, and preparation of the manuscript.

## Acknowledgments

Jingya Chen of MSD China Holding Co., Ltd., Shanghai, China, assisted literature research of the manuscript. Administrative assistance was provided by Li Qi of MSD China Holding Co., Ltd., Shanghai, China. Medical writing and editorial assistance were provided by Medjaden, Inc. This assistance was funded by MSD China.

## Conflict of interest

SC, RZ, YZ and YMZ are employees of MSD China. SR is an employee of Merck Sharp & Dohme LLC., a subsidiary of Merck & Co., Inc., Rahway, NJ, US.

The remaining authors declare that the research was conducted in the absence of any commercial or financial relationships that could be construed as a potential conflict of interest.

## Publisher’s note

All claims expressed in this article are solely those of the authors and do not necessarily represent those of their affiliated organizations, or those of the publisher, the editors and the reviewers. Any product that may be evaluated in this article, or claim that may be made by its manufacturer, is not guaranteed or endorsed by the publisher.

## Abbreviations

CI, confidence interval; DPP-4, dipeptidyl-peptidase 4; GIP, glucose-dependent insulinotropic polypeptide; GLP-1, glucagon-like peptide-1; MTT, meal tolerance test; OADs, oral anti-diabetic drugs; OGTT, oral glucose tolerance test; RCTs, randomized controlled trials; SGLT2is, sodium-glucose cotransporter 2 inhibitors; SMD, standard mean difference; T2DM, type 2 diabetes mellitus.
